# Supplementary material for: Synthesis and Biological Activity of Piperidinothiosemicarbazones Derived from Aminoazinecarbonitriles
Source: Pharmaceuticals (Basel). 2023 Sep 7;16(9):1267. doi: 10.3390/ph16091267 (PMC10535983; doi:10.3390/ph16091267)
Supplement: Supplementary file 1 [file pharmaceuticals-16-01267-s001.zip › pharmaceuticals-2542584-supplementary.pdf]

Supplementary

Synthesis and Biological Activity of Piperidinothiosemicarbazones Derived from Aminoazinecarbonitriles

Dagmara Ziembicka, Katarzyna Gobis, Małgorzata Szczesio, Ewa Augustynowicz-Kopeć, Agnieszka Głogowska, Izabela Korona-Głowniak and Krzysztof Bojanowski

Supplementary Material contains the characterization of the tested compounds. Figures **S1–S24** show NMR and ^{13}C NMR spectra of compounds **1, 4–14**.

6-morpholinopicolinonitrile (**1**)

Starting from 6-chloropicolinonitrile (5.54 g) and morpholine (4.18 mL), compound **1** was obtained as white crystals (7.56 g, 100%): m.p. 132–134°C (methanol); IR (KBr): 3098, 3069 (υ C_{Ar}-H), 2968, 2926, 2854 (υ C-H), 2230 (υ C≡N), 1595 (υ C=N), 1473, 1447 (υ C=C), 1334, 1309, 1254 (υ C-N), 1116, 1071 (υ C-O), 1027 (δ C-H), 885, 797 (γ C-H) cm^{-1}; ^{1}H NMR (500 MHz, DMSO-d_6): δ 3.47 (t, 4H, $2CH_2$, *J* = 5.0 Hz), 3.66 (t, 4H, $2CH_2$, *J* = 5.0 Hz), 7.17 (d, 1H, pyridine, *J* = 9.0 Hz), 7.22 (d, 1H, pyridine, *J* = 7.0 Hz), 7.71 (t, 1H, pyridine, *J* = 8.3 Hz) ppm; ^{13}C NMR (125 MHz, DMSO-d_6): δ 45.00 (2C), 66.20 (2C), 112.39, 118.40, 118.68, 130.64, 139.09, 159. 43 ppm; Anal. Calcd for $C_{10}H_{11}N_3O$ (189.09): C, 63.48; H, 5.86; N, 22.21; Found: C, 63.39; H, 5.94; N, 22.24.

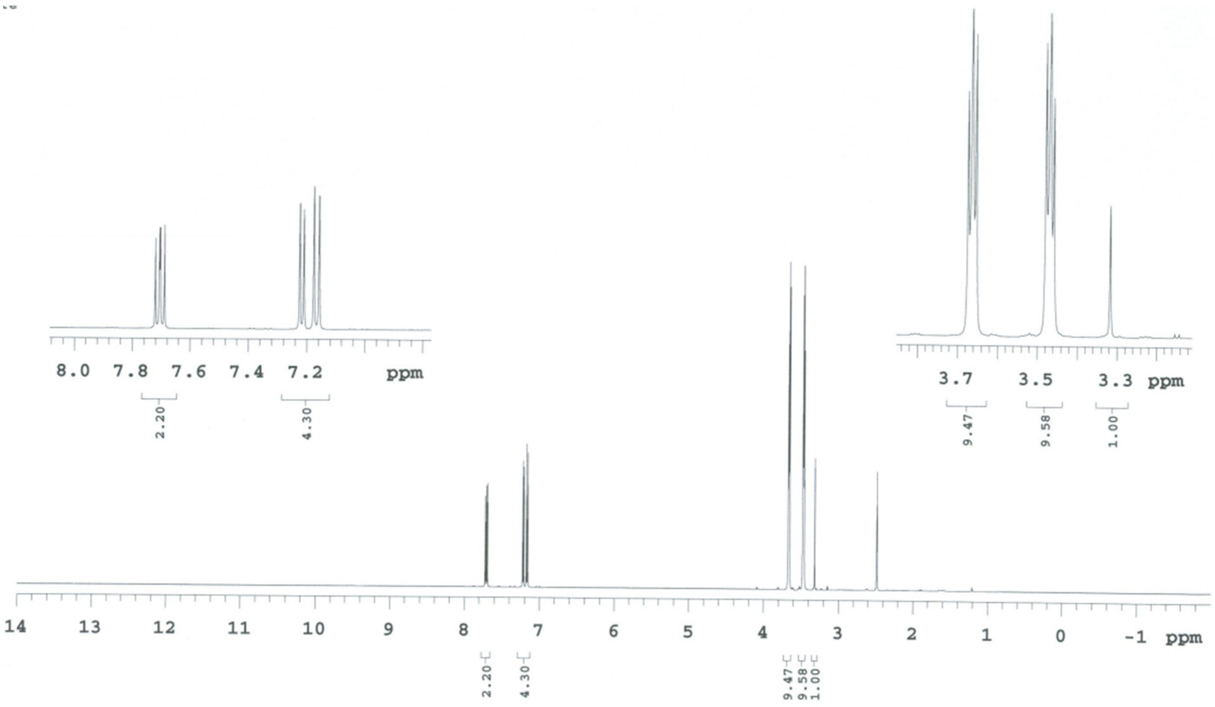

Figure S1. ^{1}H NMR spectrum (500 MHz, DMSO-d_6) of compound **1**.

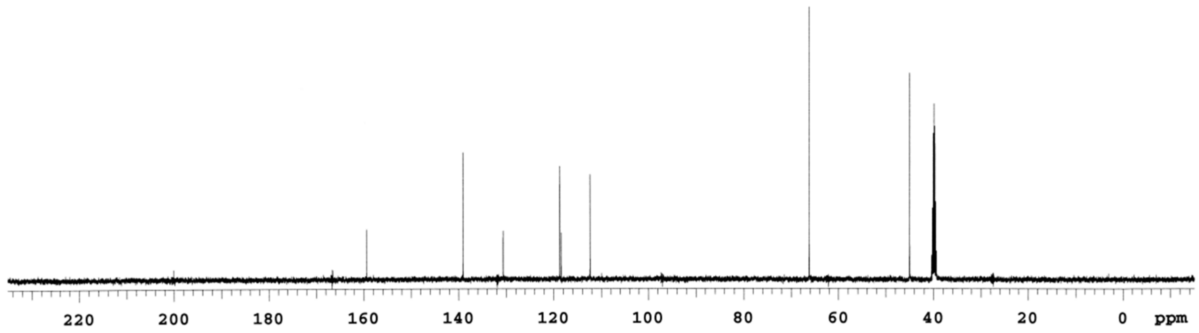

Figure S2. ^{13}C NMR spectrum (125 MHz, DMSO-d_6) of compound **1**.

The analitycal data of 6-(pyrrolidin-1-yl)picolinonitrile (**2**) and 6-(piperidin-1-yl)picolinonitrile (**3**) were described by the authors in previous work [1].

6-morpholinopyrazine-2-carbonitrile (**4**)

Starting from 6-chloropyrazine-2-carbonitrile (3.90 mL) and morpholine (4.18 mL), compound **4** was obtained as yellow crystals (6.46 g, 85%): m.p. 116–118 °C (mobile phase AcOEt:$CHCl_3$ 2:1, then recrystallization from methanol); IR (KBr): 3067 (υ C_{Ar}-H), 2982, 2912, 2871 (υ C-H), 2237 (υ C≡N), 1575 (υ C=N), 1516, 1446 (υ C=C), 1267, 1231 (υ C-N), 1118 (υ C-O), 1068 (δ C-H), 873 (γ C-H) cm^{-1}; ^1H NMR (500 MHz, DMSO-d_6): δ 3.57 (t, 4H, $2CH_2$, *J* = 5.0 Hz), 3.68 (t, 4H, $2CH_2$, *J* = 5.0 Hz), 8.31 (s, 1H, pyrazine), 8.62 (s, 1H, pyrazine) ppm; ^{13}C NMR (125 MHz, DMSO-d_6): δ 44.42 (2C), 66.03 (2C), 116.99, 126.22, 135.92, 136.59, 154.02 ppm; Anal. Calcd for $C_9H_{10}N_4O$ (190.09): C, 56.83; H, 5.30; N, 29.46; Found: C, 56.64; H, 5.16; N, 29.51.

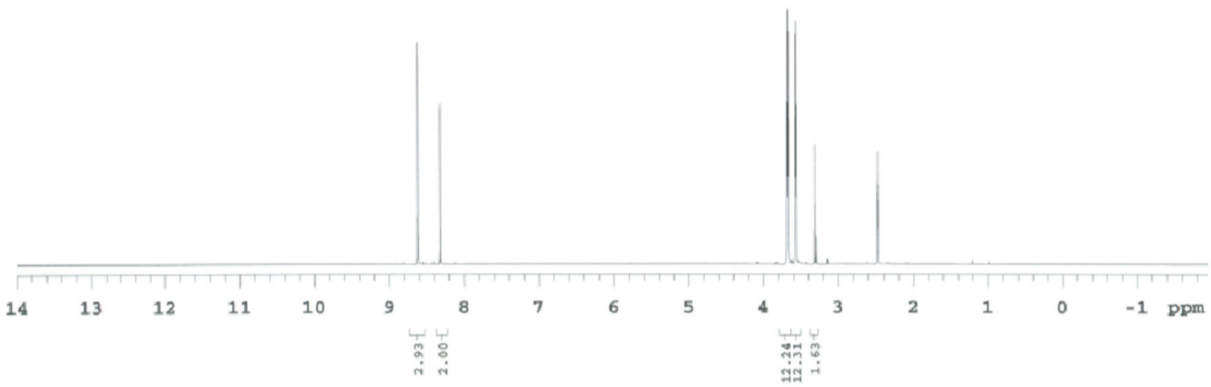

Figure S3. ^1H NMR spectrum (500 MHz, DMSO-d_6) of compound **4**.

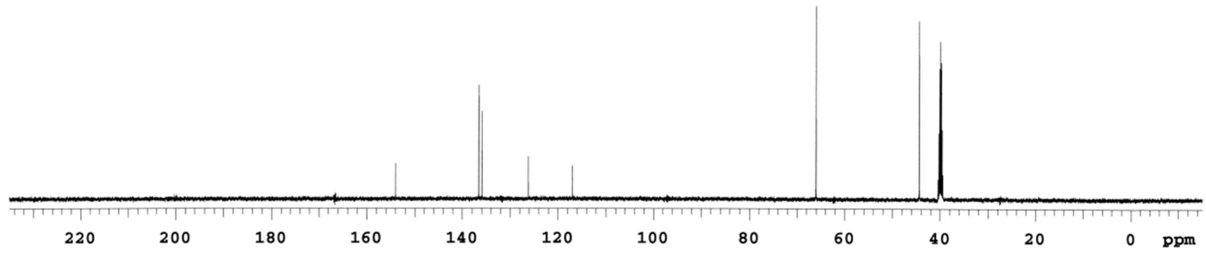

Figure S4. ^{13}C NMR spectrum (125 MHz, DMSO-d_6) of compound **4**.

6-(pyrrolidin-1-yl)pyrazine-2-carbonitrile (**5**)

Starting from 6-chloropyrazine-2-carbonitrile (3.90 mL) and pyrrolidine (4.00 mL), compound **5** was obtained as yellow crystals (4.65 g, 67%): m.p. 78–78 °C (mobile phase AcOEt:$CHCl_3$ 2:1, then recrystallization from methanol); IR (KBr): 3063 (υ C_{Ar}-H), 2979, 2951, 2863 (υ C-H), 2229 (υ C≡N), 1584 (υ C=N), 1519, 1491, 1459 (υ C=C), 1228 (υ C-N), 1161, 1059 (δ C-H), 874, 855 (γ C-H) cm^{-1}; ^1H NMR (500 MHz, DMSO-d_6): δ 1.94 (br. s, 4H, $2CH_2$), 2.48 (t, 4H, $2CH_2$, *J* = 1.8 Hz), 8.20 (s, 1H, pyrazine), 8.24 (s, 1H, pyrazine) ppm; ^{13}C NMR (125 MHz, DMSO-d_6): δ 25.22 (2C), 46.72 (2C), 117.27, 126.70, 134.41, 136.39, 151.99 ppm; Anal. Calcd for $C_9H_{10}N_4$ (174.09): C, 62.05; H, 5.79; N, 32.16; Found: C, 61.95; H, 5.48; N, 32.06.

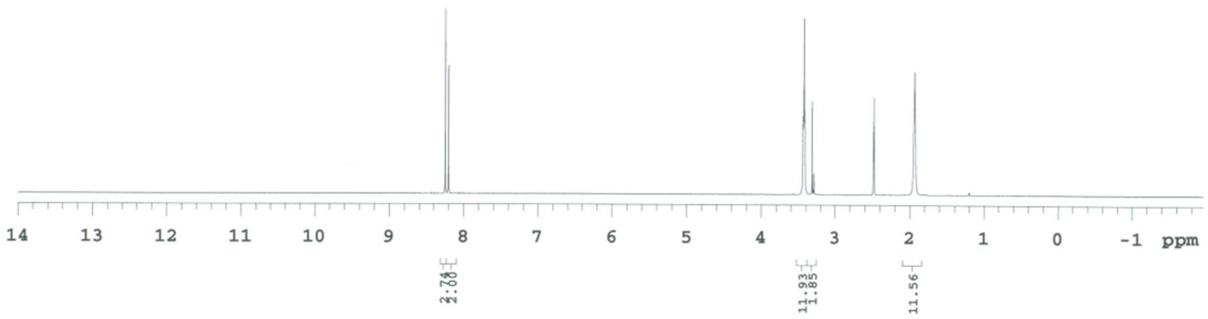

Figure S5. ^1H NMR spectrum (500 MHz, DMSO-d_6) of compound **5**.

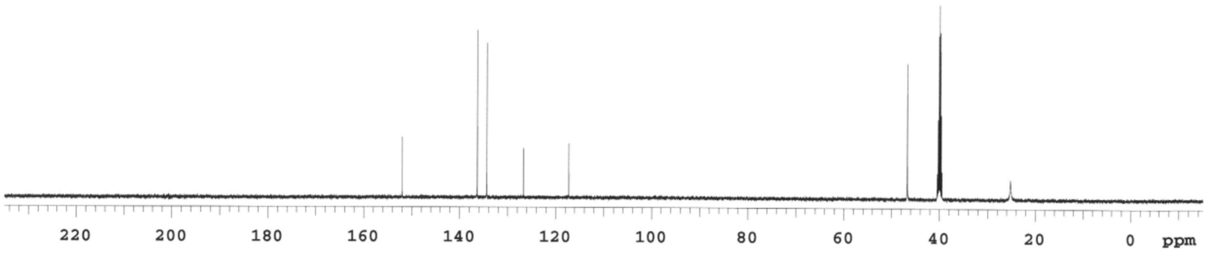

Figure S6. ^{13}C NMR spectrum (125 MHz, DMSO-d_6) of compound **5**.

6-(piperidin-1-yl)pyrazine-2-carbonitrile (**6**)

Starting from 6-chloropyrazine-2-carbonitrile (3.9 mL) and pyrrolidine (4.75 mL), compound **6** was obtained as yellow crystals (7.50 g, 100%): m.p. 68–69 °C (mobile phase AcOEt:$CHCl_3$ 2:1, then recrystallization from methanol); IR (KBr): 3081, 3008 (υ C_{Ar}-H), 2948, 2858 (υ C-H), 2227 (υ C≡N), 1579 (υ C=N), 1516, 1451, 1417 (υ C=C), 1278, 1239, 1208 (υ C-N), 1136, 1041 (δ C-H), 850 (γ C-H) cm^{-1}; ^1H NMR (500 MHz, DMSO-d_6): δ 1.52–1.55 (m, 4H, $2CH_2$), 1.60–1.63 (m, H, CH_2), 3.60 (t, 4H, $2CH_2$, *J* = 5.3 Hz), 8.20 (s, 1H, pyrazine), 8.60 (s, 1H, pyrazine) ppm; ^{13}C NMR (125 MHz, DMSO-d_6): δ 24.32, 25.34 (2C), 45.16 (2C), 117.11, 126.32, 134.74, 136.43, 153.71 ppm; Anal. Calcd for $C_{10}H_{12}N_4$ (188.11): C, 63.81; H, 6.43; N, 29.77; Found: C, 63.57; H, 6.68; N, 29.95.

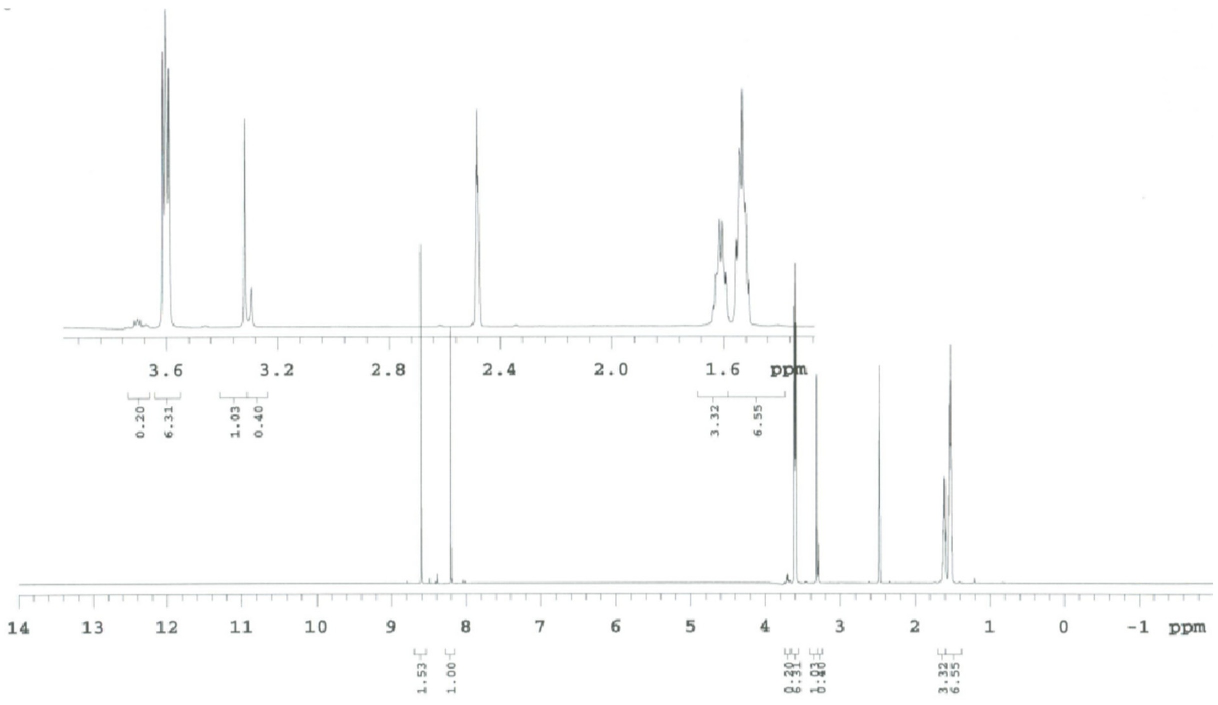

Figure S7. ^{1}H NMR spectrum (500 MHz, DMSO-d_6) of compound **6**.

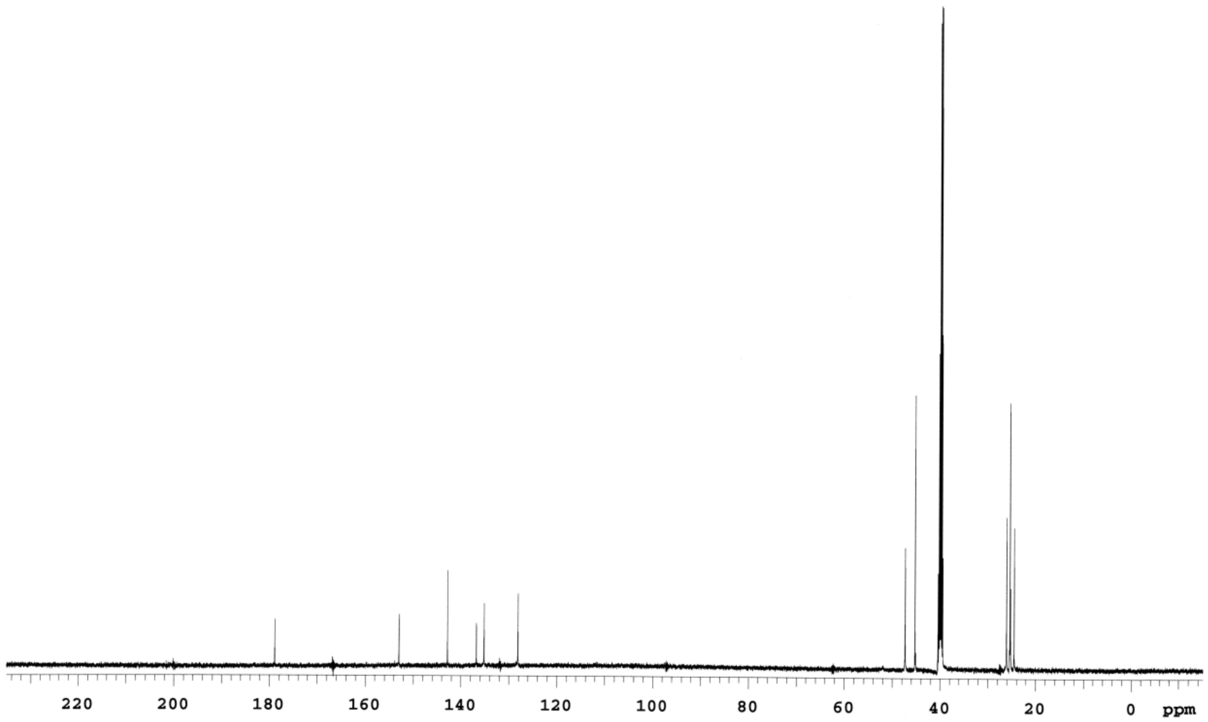

Figure S8. ^{13}C NMR spectrum (125 MHz, DMSO-d_6) of compound **6**.

The analitycal data of 4-morpholinopicolinonitrile (**DMK-4**) and 4-(pyrrolidin-1-yl)picolinonitrile (**DMK-3**) were described by the authors in previous work [2].

4-(piperidin-1-yl)picolinonitrile (**7**)

Starting from 4-chloropicolinonitrile (5.54 g) and piperidine (4.75 mL), compound **7** was obtained as white crystals (6.91 g, 92%): m.p. 79–81°C (mobile phase AcOEt:$CHCl_3$ 2:1, then recrystallization from methanol); IR (KBr): 3002 (υ C_{Ar}-H), 2916, 2851 (υ C-H), 2233 (υ C≡N), 1596 (υ C=N), 1500, 1442 (υ C=C), 1266, 1215 (υ C-N), 1023 (δ C-H), 817 (γ C-H) cm^{-1}; ^1H NMR (500 MHz, DMSO-d_6): δ 1.52–1.55 (m, 4H, $2CH_2$), 1.61–1.63 (m, 2H, CH_2), 3.43 (t, 4H, $2CH_2$, *J* = 5.5 Hz), 7.02 (dd, 1H, pyridine, J_1 = 9.0 Hz, J_2 = 3.0 Hz), 7.45 (d, 1H, pyridine, J = 3.0 Hz), 8.18 (d, 1H, pyridine, *J* = 6.0 Hz) ppm; ^{13}C NMR (125 MHz, DMSO-d_6): δ 24.27, 25.05 (2C), 46.89 (2C), 110.72, 113.40, 118.82, 133.86, 151.38, 154.46 ppm; Anal. Calcd for $C_{11}H_{13}N_3$ (187.11): C, 70.56; H, 7.00; N, 22.44; Found: C, 70.62; H, 7.03; N, 22.31.

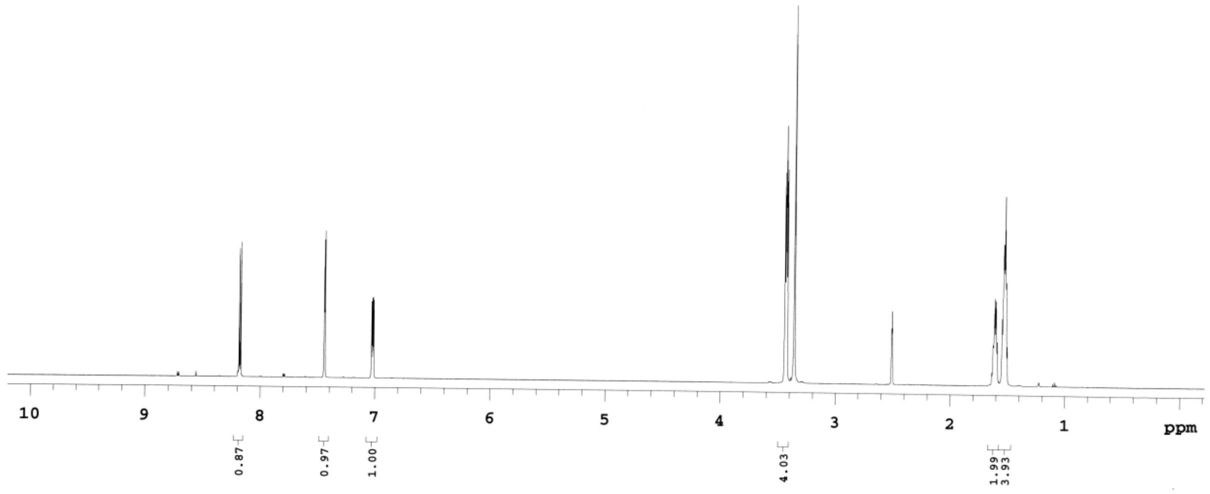

Figure S9. ^1H NMR spectrum (500 MHz, DMSO-d_6) of compound **7**.

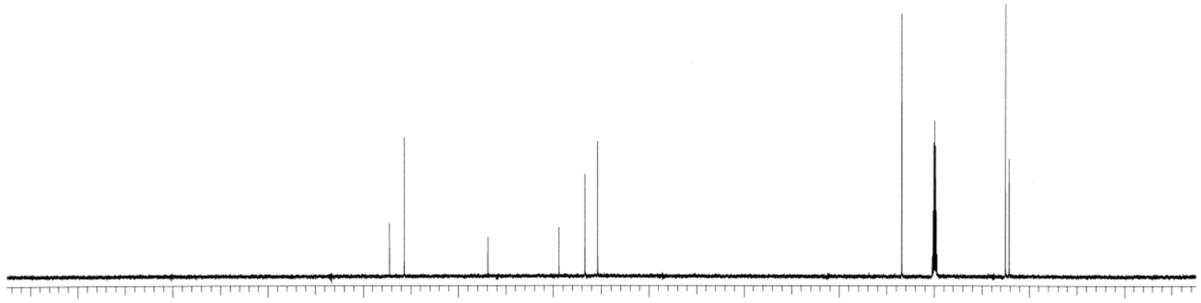

Figure S10. ^{13}C NMR spectrum (125 MHz, DMSO-d_6) of compound **7**.

6-morpholino-*N′*-(piperidine-1 carbonothioyl)picolinohydrazonamide (**8**)

Starting from 6-morpholinopicolinonitrile (0.378 g), the title compound **8** was obtained as yellow crystals (0.447 g, 68%): m.p. 159–160 °C (benzene, anhydrous ethanol); IR (KBr): 3390, 3206 (υ N-H), 3093, 3008 (υ C_{Ar}-H), 2921, 2849 (υ C-H), 1671 (υ C=N), 1601, 1559 (δ N-H), 1478, 1430 (υ C=C), 1338, 1317, 1248 (υ C-N), 1113 (υ C-O), 1028 (δ C-H), 880, 871, 791 (γ C-H) cm^{-1}; ^1H NMR (500 MHz, DMSO-d_6): δ 1.45 (br. s, 4H, $2CH_2$), 1.57–1.59 (m, 2H, CH_2), 3.62–3.63 (m, 4H, $2CH_2$), 3.71 (t, 4H, $2CH_2$, *J* = 4.8 Hz), 3.84 (t, 4H, $2CH_2$, *J* = 5.3 Hz), 7.09 (d, 1H, pyridine, *J* = 8.5 Hz), 7.42 (d, 1H, pyridine, *J* = 7.5 Hz), 7.61 (s, 1H, NH), 7.77 (t, 1H, pyridine, *J* = 8.0 Hz), 8.28 (br. s, 1H, NH), 12.78 (s, 1H, NH) ppm; ^{13}C NMR (125

MHz, DMSO-d_6): δ 25.30, 26.10 (2C), 45.29 (2C), 47.35 (2C), 66.27 (2C), 110.58, 110.87, 139.56, 142.19, 144.20, 158.32, 178.58 ppm; Anal. Calcd for $C_{16}H_{24}N_6OS$ (348.17): C, 55.15; H, 6.94; N, 24.12; Found: C, 55.33; H, 6.78; N, 23.94.

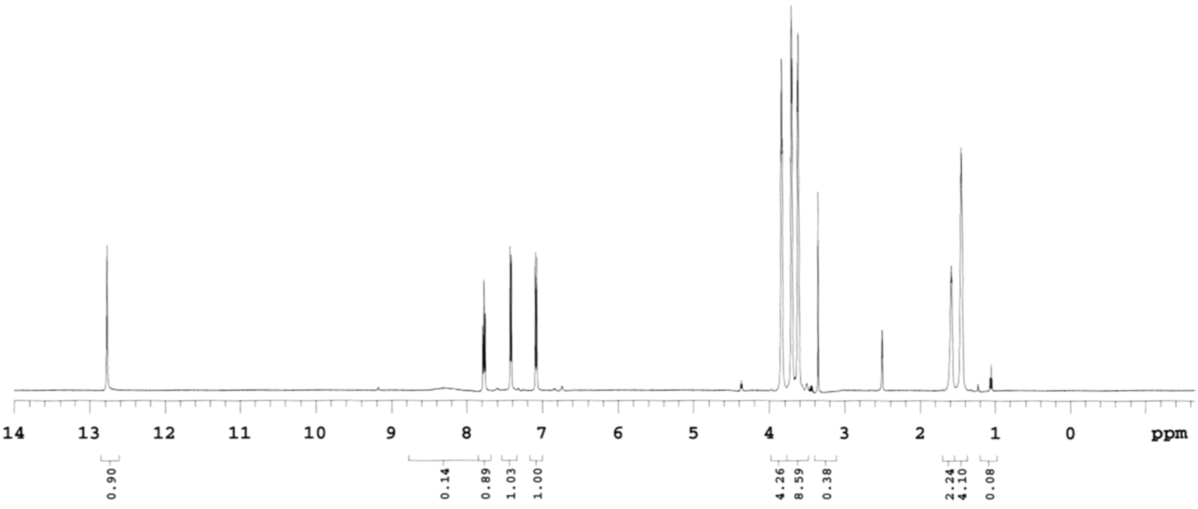

Figure S11. ^{1}H NMR spectrum (500 MHz, DMSO-d_6) of compound **8**.

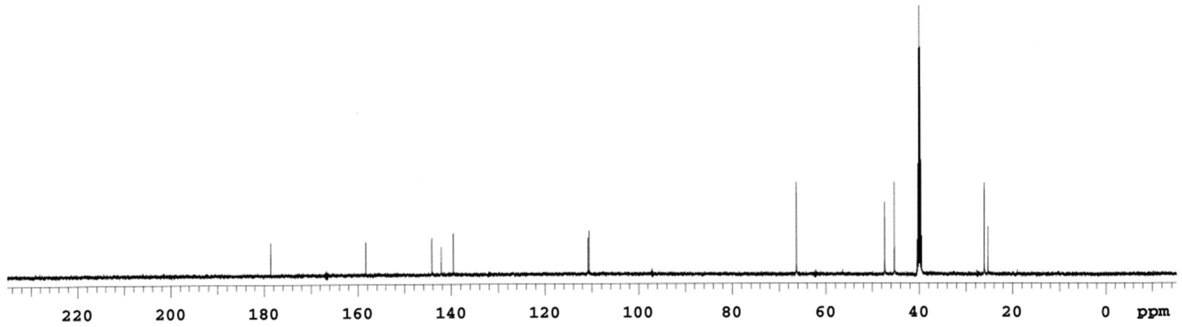

Figure S12. ^{13}C NMR spectrum (125 MHz, DMSO-d_6) of compound **8**.

N′-(piperidine-1-carbonothioyl)-6-(pyrrolidin-1-yl)picolinohydrazonamide (**9**)

Starting from 6-(pyrrolidin-1-yl)picolinonitrile (0.346 g), the title compound **9** was obtained as yellow crystals (0.545 g, 82%): m.p. 171–172 °C (mobile phase AcOEt:$CHCl_3$ 1:5); IR (KBr): 3405, 3211 (υ N-H), 3065 (υ C_{Ar}-H), 2929, 2852 (υ C-H), 1666 (υ C=N), 1604, 1555 (δ N-H), 1504, 1469, 1420 (υ C=C), 1373, 1339, 1307, 1246 (υ C-N), 1163 (υ C=S), 1024 (δ C-H), 884, 789 (γ C-H) cm^{-1}; ^{1}H NMR (500 MHz, DMSO-d_6): δ 1.45 (br. s, 4H, $2CH_2$), 1.57-1.59 (m, 2H, CH_2), 1.97 (br. s, 4H, $2CH_2$), 3.48 (br. s, 4H, $2CH_2$), 3.84 (t, 4H, $2CH_2$, *J* = 5.0 Hz), 6.68 (d, 1H, pyridine, *J* = 8.5 Hz), 7.29 (d, 1H, pyridine, *J* = 7.5 Hz), 7.51 (br. s, 1H, NH), 7.68 (t, 1H, pyridine, *J* = 8.0 Hz), 8.25 (br. s, 1H, NH), 12.72 (s, 1H, NH) ppm; ^{13}C NMR (125 MHz, DMSO-d_6): δ 25.32, 25.40 (2C), 26.11 (2C), 46.80 (2C), 47.33 (2C), 108.56, 110.57, 138.62, 142.40, 144.69, 156.09, 178.49 ppm; Anal. Calcd for $C_{16}H_{24}N_6S$ (332.18): C, 57.80; H, 7.28; N, 25.28; Found: C, 57.52; H, 7.42; N, 25.31.

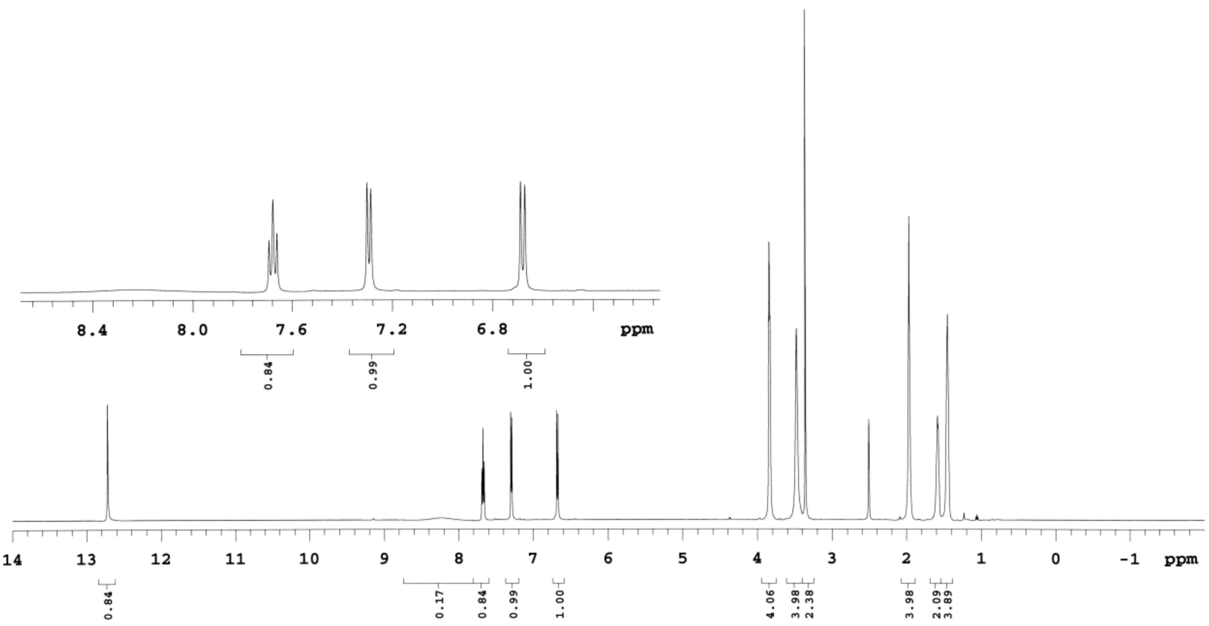

Figure S13. ^{1}H NMR spectrum (500 MHz, DMSO-d_6) of compound **9**.

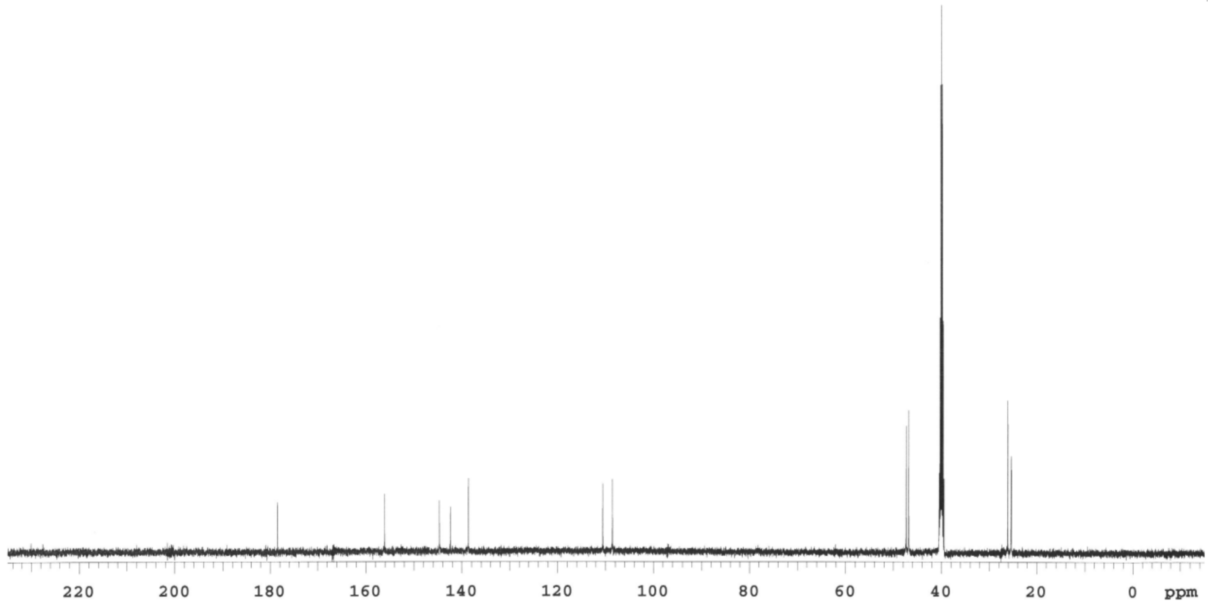

Figure S14. ^{13}C NMR spectrum (125 MHz, DMSO-d_6) of compound **9**.

6-(piperidin-1-yl)-*N*′-(piperidine-1-carbonothioyl)picolinohydrazonamide (**10**)

Starting from 6-(piperidin-1-yl)picolinonitrile (0.374 g), the title compound **10** was obtained as yellow crystals (0.654 g, 95%): m.p. 150–152 °C (anhydrous ethanol); IR (KBr): 3408, 3222, 3118 (υ N-H), 3026 (υ C_{Ar}-H), 2934, 2851 (υ C-H), 1671 (υ C=N), 1604, 1558 (δ N-H), 1477, 1428 (υ C=C), 1341, 1313, 1248 (υ C-N), 1184 (υ C=S), 1108, 1025 (δ C-H), 887, 795 (γ C-H) cm^{-1}; ^{1}H NMR (500 MHz, DMSO-d_6): δ 1.45-1.54 (m, 8H, $4CH_2$), 1.57-1.63 (m, 4H, $2CH_2$), 3.67 (t, 4H, $2CH_2$, *J* = 5.3 Hz), 3.84 (t, 4H, $2CH_2$, *J* = 5.3 Hz), 7.06 (d, 1H, pyridine, *J* = 8.5 Hz), 7.32 (d, 1H, pyridine, *J* = 7.0 Hz), 7.51 (br. s, 1H, NH), 7.69 (t, 1H, pyridine, *J* = 8.0 Hz), 8.23 (br. s, 1H, NH), 12.75 (s, 1H, NH) ppm; ^{13}C NMR (125 MHz, DMSO-d_6): δ 24.76, 25.32, 25.43 (2C), 26.11 (2C), 45.90 (2C), 47.33 (2C), 109.38, 110.80, 139.29, 142.25, 144.45, 158.00, 178.56 ppm; Anal. Calcd for $C_{17}H_{26}N_6S$ (346.19): C, 58.93; H, 7.56; N, 24.25; Found: C, 58.70; H, 7.44; N, 24.34.

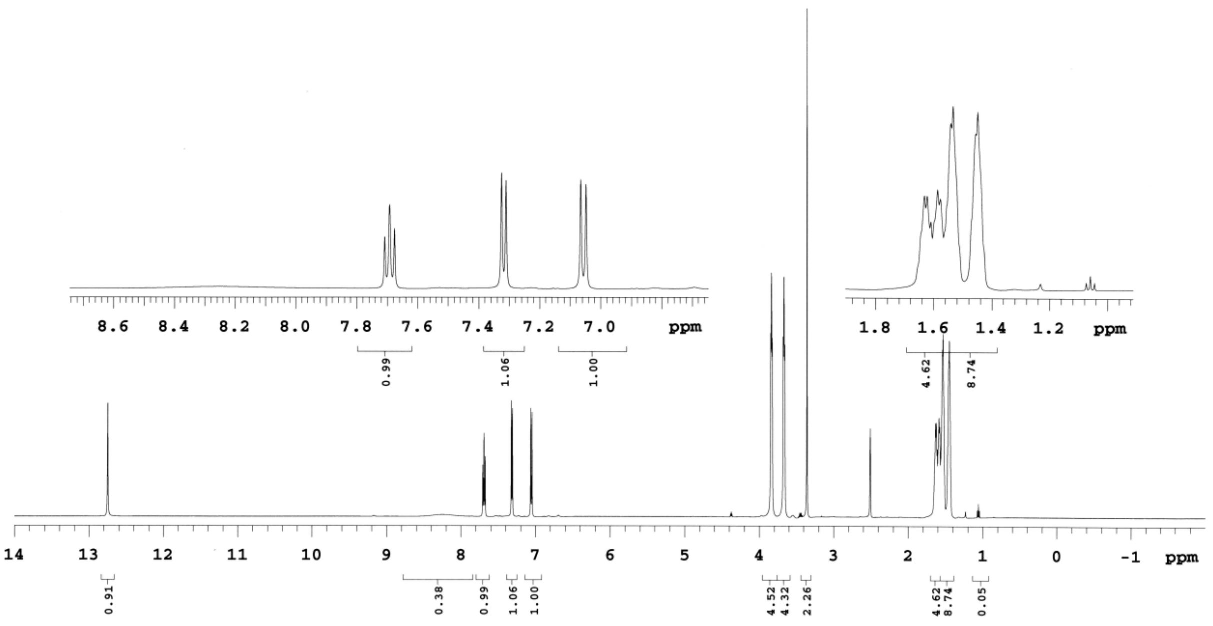

Figure S15. ^{1}H NMR spectrum (500 MHz, DMSO-d_6) of compound **10**.

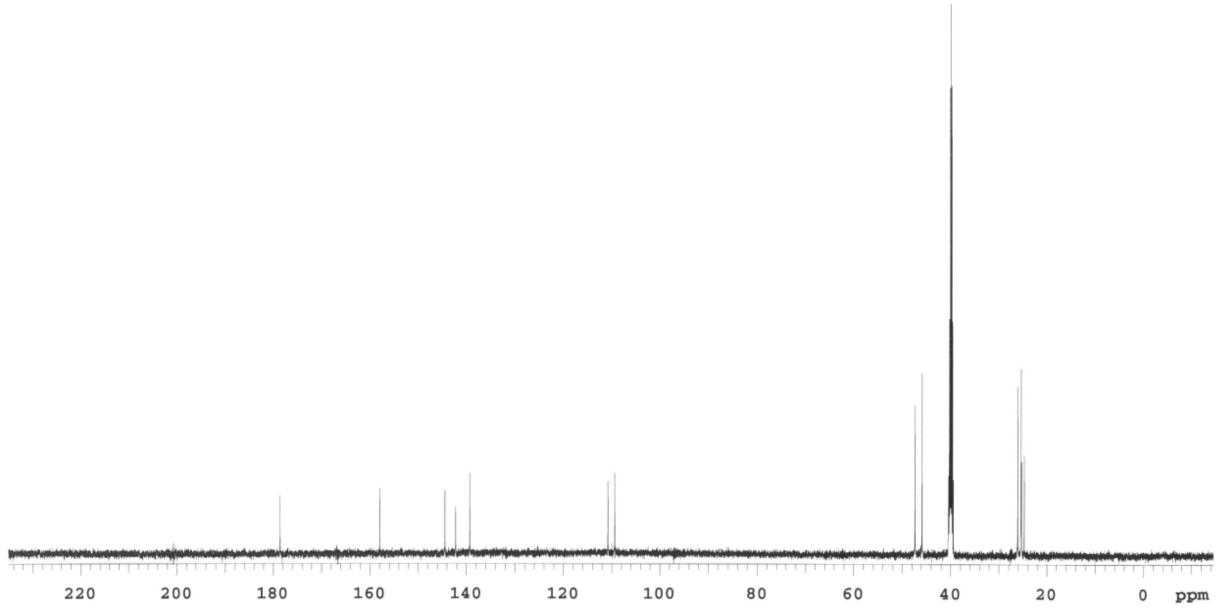

Figure S16. ^{13}C NMR spectrum (125 MHz, DMSO-d_6) of compound **10**.

6-morpholino-*N′*-(piperidine-1-carbonothioyl)pyrazine-2-carbohydrazonamide (**11**)

Starting from 6-morpholinopyrazine-2-carbonitrile (0.571 g), the title compound **11** was obtained as yellow crystals (0.671 g, 64%): m.p. 170–171 °C (mobile phase AcOEt:$CHCl_3$ 1:2, then recrystallization from ethanol); IR (KBr): 3422, 3300, 3166 (υ N-H), 2926, 2851 (υ C-H), 1672 (υ C=N), 1576 (δ N-H), 1528, 1470, 1448 (υ C=C), 1299, 1261, 1243 (υ C-N), 1116 (υ C-O), 1067, 1000 (δ C-H), 874 (γ C-H) cm^{-1}; ^{1}H NMR (500 MHz, DMSO-d_6): δ 1.43 (br. s, 4H, $2CH_2$), 1.56–1.58 (m, 2H, CH_2), 3.68–3.71 (m, 8H, $4CH_2$), 3,82 (t, 4H, $2CH_2$, J = 5.3 Hz) 6.79 (s, 1H, NH), 7.80 (br. s, 1H, NH), 8.51 (s, 1H, pyrazine), 8.53 (s, 1H, pyrazine), 12.77 (s, 1H, NH) ppm; ^{13}C NMR (125 MHz, DMSO-d_6): δ 25.26, 26.10 (2C), 44.58 (2C), 47.36 (2C), 66.08 (2C), 129.29, 135.26, 136.76, 142.61, 153.18, 178.89 ppm; Anal. Calcd for $C_{15}H_{23}N_7OS$ (349.17): C, 51.55; H, 6.63; N, 28.06; Found: C, 51.90; H, 6.48; N, 28.06.

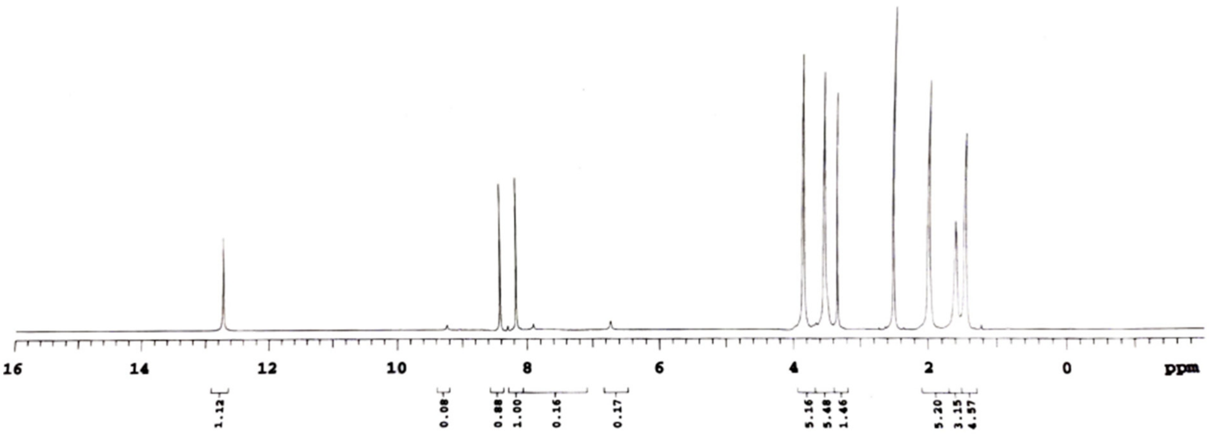

Figure S17. ^{1}H NMR spectrum (500 MHz, DMSO-d_6) of compound **11**.

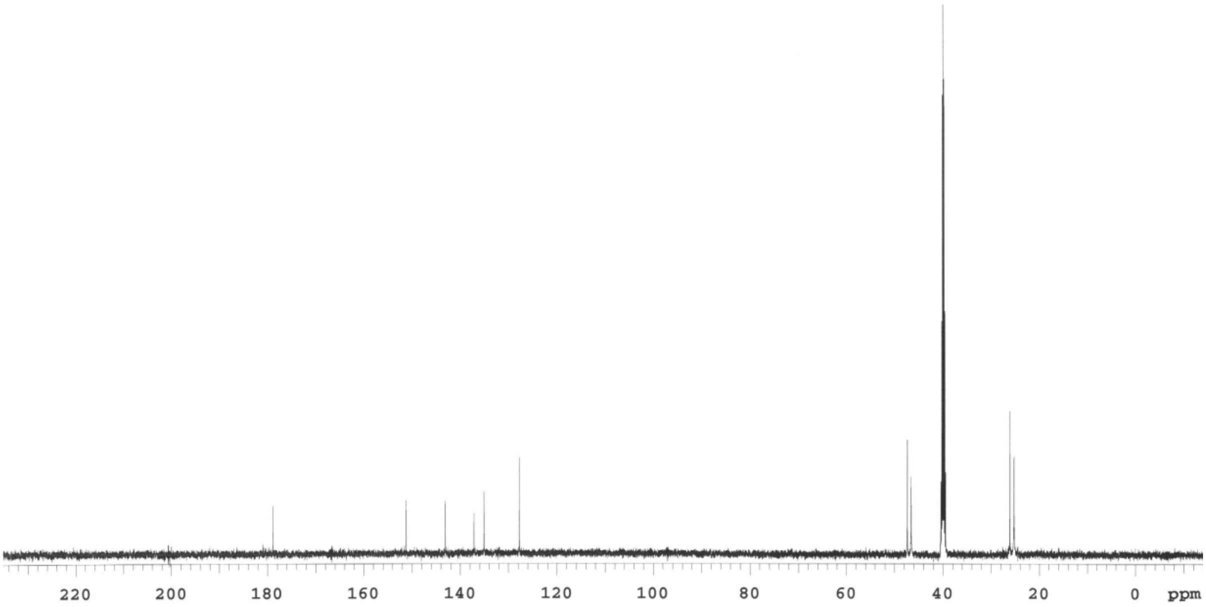

Figure S18. ^{13}C NMR spectrum (125 MHz, DMSO-d_6) of compound **11**.

N'-(piperidine-1-carbonothioyl)-6-(pyrrolidin-1-yl)pyrazine-2-carbohydrazonamide (**12**)

Starting from 6-(pyrrolidin-1-yl)pyrazine-2-carbonitrile (0.523 g), the title compound **12** was obtained as yellow crystals (0.334 g, 33%): m.p. 163–164 °C (methanol–water 1:1); IR (KBr): 3415, 3212 (υ N-H), 3054 (υ C_{Ar}-H), 2920, 2853 (υ C-H), 1661 (υ C=N), 1599 (δ N-H), 1531, 1425 (υ C=C), 1343, 1291, 1245 (υ C-N), 1138 (υ C=S), 1099, 1024 (δ C-H), 886, 848, 828 (γ C-H) cm^{-1}; ^{1}H NMR (500 MHz, DMSO-d_6): δ 1.44 (br. s, 4H, $2CH_2$), 1.58 (br. s, 2H, CH_2), 1.98 (br. s, 4H, $2CH_2$), 3.52 (br. s, 4H, $2CH_2$), 3.84 (br. s, 4H, $2CH_2$), 6.71 (s, 1H, NH), 7.89 (s, 1H, NH), 8.17 (s, 1H, pyrazine), 8.42 (s, 1H, pyrazine), 12.73 (s, 1H, NH) ppm; ^{13}C NMR (125 MHz, DMSO-d_6): δ 25.28 (3C), 26.11 (2C), 46.55 (2C), 47.34 (2C), 127.74, 135.05, 137.15, 143.10, 151.19, 178.84 ppm; Anal. Calcd for $C_{15}H_{23}N_7S$ (333.17): C, 54.03; H, 6.95; N, 29.40; Found: C, 54.12; H, 6.57; N, 29.10.

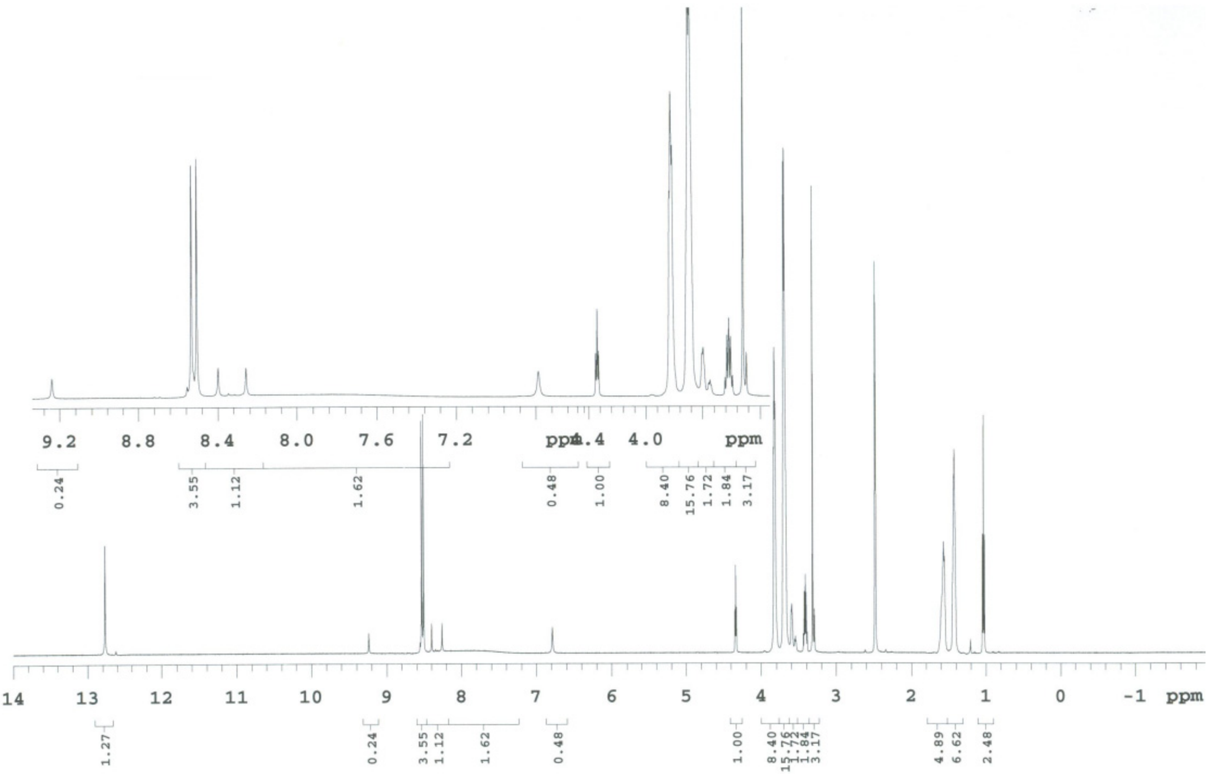

Figure S19. ^{1}H NMR spectrum (500 MHz, DMSO-d_6) of compound **12**.

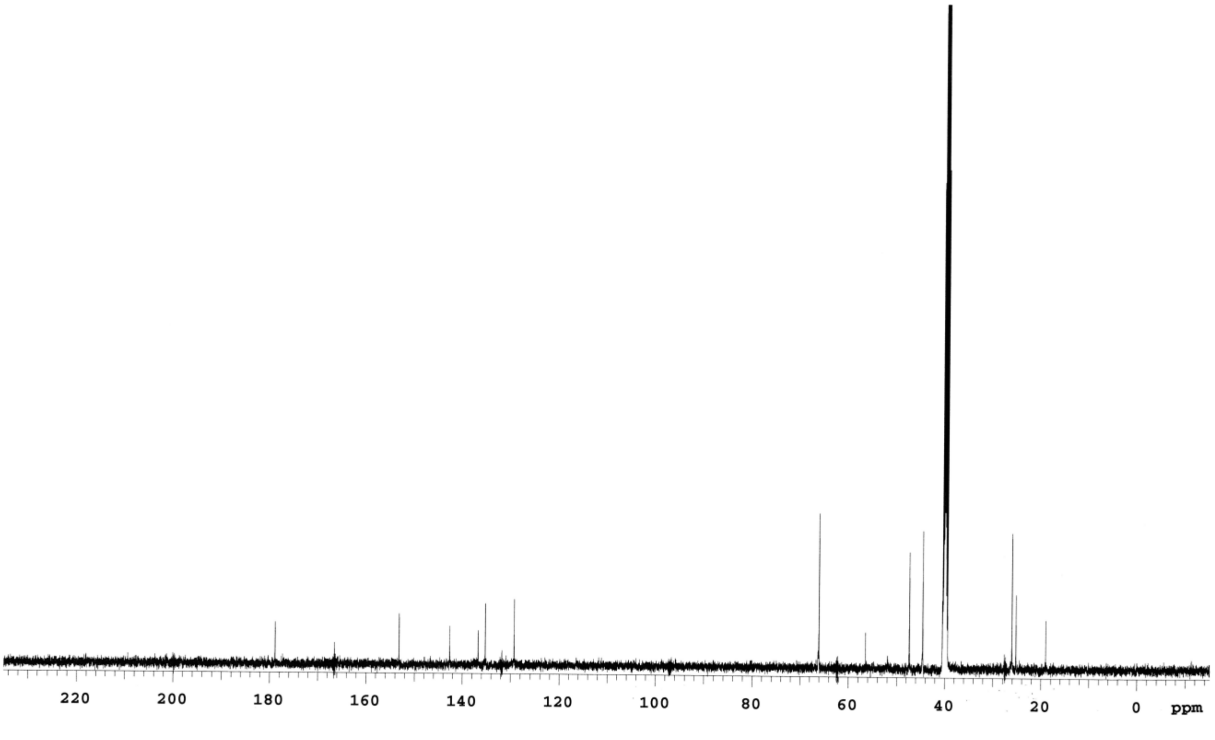

Figure S20. ^{13}C NMR spectrum (125 MHz, DMSO-d_6) of compound **12**.

6-(piperidin-1-yl)-*N′*-(piperidine-1-carbonothioyl)pyrazine-2-carbohydrazonamide (**13**)

Starting from 6-(piperidin-1-yl)pyrazine-2-carbonitrile (0.565 g), the title compound **13** was obtained as yellow crystals (0.594 g, 57%): m.p. 171–174 °C (anhydrous ethanol); IR (KBr): 3388 (υ N-H), 3093 (υ C_{Ar}-H), 2924, 2847 (υ C-H), 1674 (υ C=N), 1584 (δ N-H), 1526, 1473, 1424 (υ C=C), 1310, 1271, 1234 (υ C-N), 1138 (υ C=S), 1053, 1023 (δ C-H), 886, 854, 835 (γ C-H) cm^{-1}; ^{1}H NMR (500 MHz, DMSO-d_6): δ 1.43 (br. s, 4H, $2CH_2$), 1.54–1.57 (m, 6H, $3CH_2$), 1.62–1.63 (br. s, 2H, CH_2), 3.71 (t, 4H, $2CH_2$, *J* = 5.3 Hz), 3.82 (t, 4H, $2CH_2$, *J* = 5.3 Hz), 6.72 (s, 1H, NH), 7.70 (br. s, 1H, NH), 8.41 (s, 1H, pyrazine), 8.52 (s, 1H, pyrazine), 12.75 (s, 1H, NH) ppm; ^{13}C NMR (125 MHz, DMSO-d_6): δ 24.52, 25.27, 25.41 (2C), 26.11 (2C), 45.30 (2C), 47.36 (2C), 128.14, 135.18, 136.79, 142.84, 152.95, 178.88 ppm; Anal. Calcd for $C_{16}H_{25}N_7S$ (347.19): C, 55.30; H, 7.25; N, 28.22; Found: C, 55.40; H, 7.07; N, 28.01.

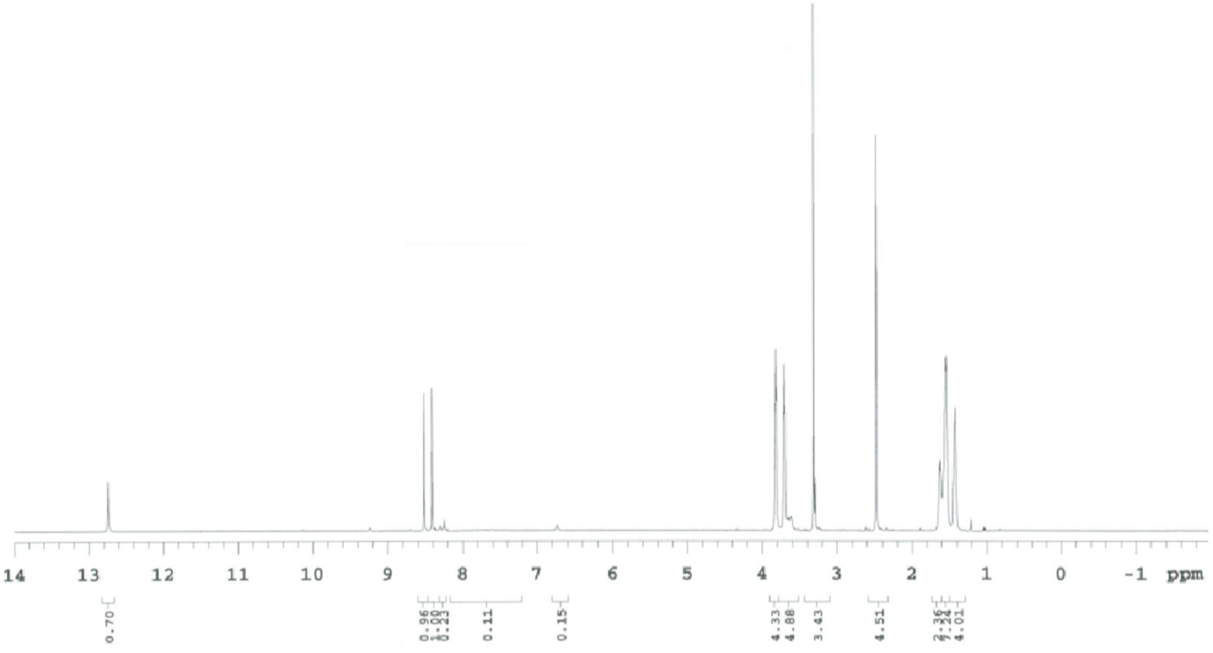

Figure S21. ^{1}H NMR spectrum (500 MHz, DMSO-d_6) of compound **13**.

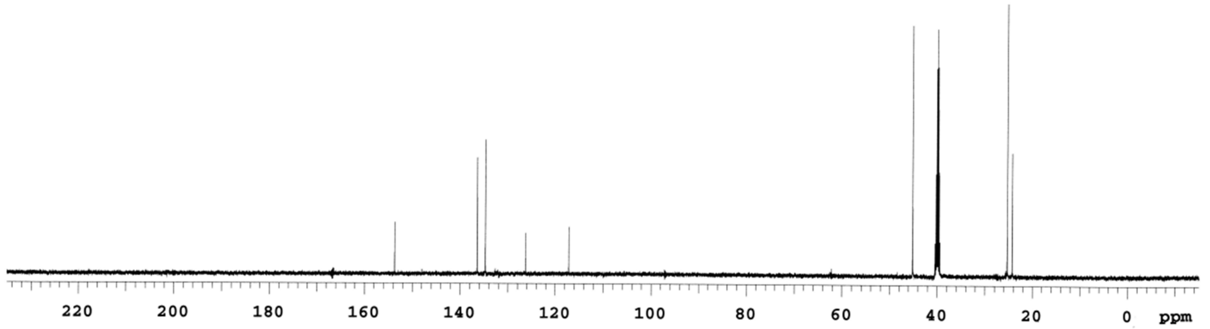

Figure S22. ^{13}C NMR spectrum (125 MHz, DMSO-d_6) of compound **13**.

The analitycal data of 4-morpholino-*N′*-(piperidine-1 carbonothioyl)picolinohydrazonamide (**DMK-20**) and *N′*-(piperidine-1-carbonothioyl)-4-(pyrrolidin-1-yl)picolinohydrazonamide (**DMK-16**) were described by the authors in previous work [2].

4-(piperidin-1-yl)-*N′*-(piperidine-1-carbonothioyl)picolinohydrazonamide (**14**)

Starting from 4-(piperidin-1-yl)picolinonitrile (0.375 g), the title compound **14** was obtained as yellow crystals (0.495 g, 71%): m.p. 188–190 °C (anhydrous ethanol); IR (KBr): 3403, 3240 (υ N-H), 3067 (υ C_{Ar}-H), 2931, 2845 (υ C-H), 1665 (υ C=N), 1606, 1577 (δ N-H), 1483, 1421 (υ C=C), 1378, 1356, 1309, 1241 (υ C-N), 1123, 1097 (δ C-H), 884, 852, 822 (γ C-H) cm^{-1}; ^{1}H NMR (500 MHz, DMSO-d_6): δ 1.43-1.44 (m, 4H, $2CH_2$), 1.55-1.57 (m, 6H, $3CH_2$), 1.60-1.62 (m, 2H, CH_2), 3.46 (t, 4H, $2CH_2$, *J* = 5.5 Hz), 3.81 (t, 4H, $2CH_2$, *J* = 5.3 Hz), 6.90 (dd, 1H, pyridine, J_1 = 8.5 Hz, J_2 = 4.0 Hz), 7.38 (br. s, 1H, NH), 7.52 (d, 1H, pyridine, *J* = 2.0 Hz), 8.21 (d, 1H, pyridine, *J* = 6.5 Hz), 8.41 (br. s, 1H, NH), 12.56 (s, 1H, NH) ppm; ^{13}C NMR (125 MHz, DMSO-d_6): δ 24.35, 25.20 (2C), 25.31, 26.12 (2C), 47.02 (2C), 47.30 (2C), 105.74, 109.45, 145.00, 145.13, 150.17, 155.50, 178.37 ppm; Anal. Calcd for $C_{17}H_{26}N_6S$ (346.19): C, 58.93; H, 7.56; N, 24.25; Found: C, 58.73; H, 7.61; N, 24.24.

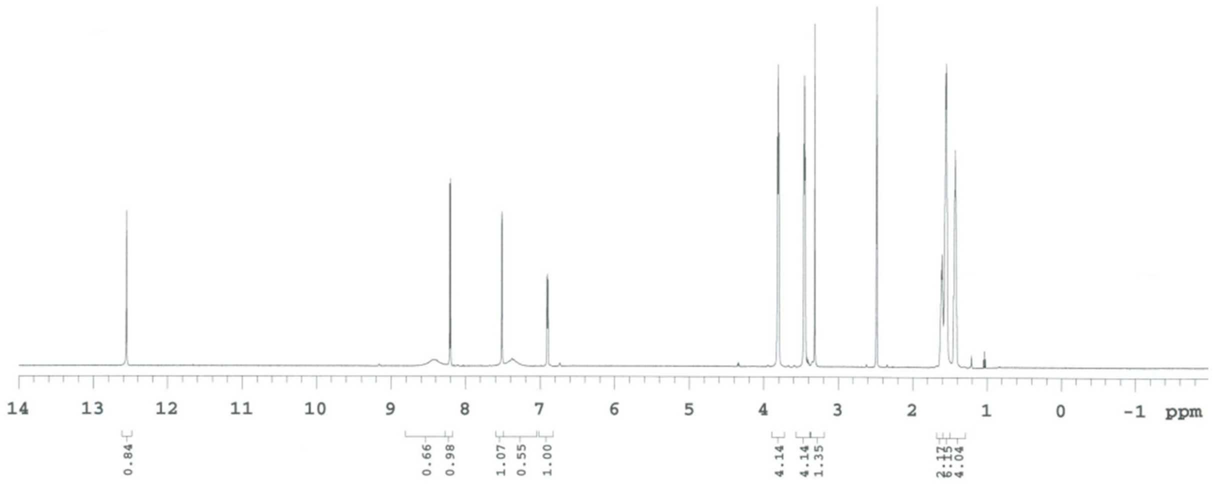

Figure S23. ^{1}H NMR spectrum (500 MHz, DMSO-d_6) of compound **14**.

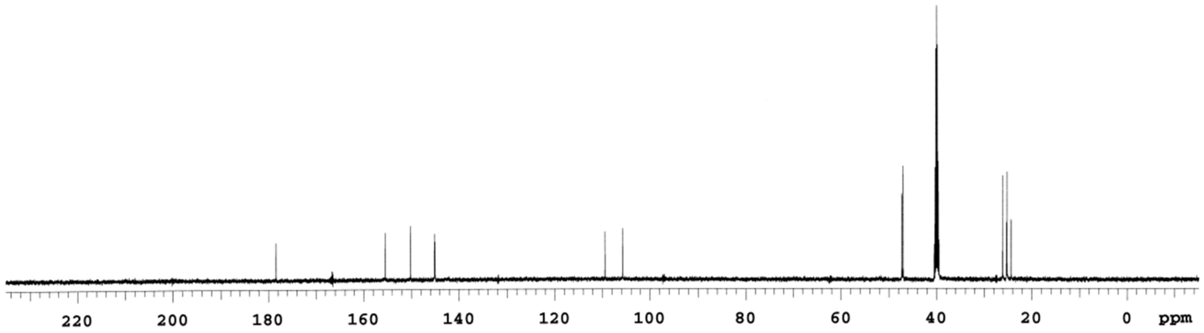

Figure S24. ^{13}C NMR spectrum (125 MHz, DMSO-d_6) of compound **14**.

References

1. Ziembicka, D.; Gobis, K.; Szczesio, M.; Olczak, A.; Augustynowicz-Kopeć, E.; Głogowska, A.; Korona-Głowniak, I.; Bojanowski, K. Synthesis and structure-activity relationship of 2,6-disubstituted thiosemicarbazone derivatives of pyridine as potential antituberculosis agents. *Materials* **2023**, *16*, 448.
2. Krause, M.; Foks, H.; Ziembicka, D.; Augustynowicz-Kopeć, E.; Głogowska, A.; Korona-Głowniak, I.; Bojanowski, K.; Siluk, D.; Gobis, K. 4-substituted picolinohydrazonamides as a new class of potential antitubercular agents. *Eur. J. Med. Chem.* **2020**, *190*, 112106.
